# A network meta-analysis for neoadjuvant and adjuvant treatments for resectable squamous cell carcinoma of esophagus

**DOI:** 10.1038/s41598-021-86102-8

**Published:** 2021-03-24

**Authors:** Yunpeng Zhao, Yongqiang Wang, Lei Shan, Chuanliang Peng, Wenhao Zhang, Xiaogang Zhao

**Affiliations:** Department of Thoracic Surgery, The Second Hospital of Shandong University, Jinan, Shandong Province China

**Keywords:** Cancer therapy, Chemotherapy, Radiotherapy

## Abstract

The optimal treatment for resectable esophageal squamous cell carcinoma (ESCC) is still a debatable point; however, randomized trials for strategies including neoadjuvant or adjuvant chemotherapy (CT), radiotherapy, or chemoradiotherapy (CRT) are not always available. This network meta-analysis aimed to identify an effective approach through indirect comparisons. An extensive literature search comparing multimodality treatment and surgery was performed, and a network meta-analysis was conducted with the frequentist method. Twenty-three trials including a total of 3636 ESCC patients were included. Neoadjuvant CRT and neoadjuvant CT, which were recommended by most guidelines for esophageal cancer, were associated with an overall survival advantage compared with surgery alone (HR = 0.43, 95% CI 0.26–0.73; HR = 0.71, 95% CI 0.32–1.59). A statistically significant survival benefit from neoadjuvant CRT compared with neoadjuvant CT could not be demonstrated in our study (HR = 0.61, 95% CI 0.32–1.17, *P* = 0.08). Our network meta-analysis showed that both neoadjuvant CRT and neoadjuvant CT were effective in improving the survival of patients with ESCC. Individual clinical decisions need further study in the future.

## Introduction

Esophageal carcinoma has the eighth highest incidence and is the sixth leading cause of carcinoma-related deaths worldwide^1,2^. Esophageal squamous cell carcinoma (ESCC) is the most frequent histological subtype^3^. The 5-year survival rate of patients with ESCC is still low^4^.

Surgery is still a potentially curative treatment; however, neoadjuvant or adjuvant therapies have been proven to improve survival^5^, including chemotherapy (CT), radiotherapy (RT), or synchronous chemoradiotherapy (CRT) performed before or after surgery^6,7^. Multimodality treatments show overall survival (OS) differences in a large number of trials conducted in the past, even though some conclusions were controversial; for example, preoperative CT followed by surgery was demonstrated to improve survival in one trial^8^, while another trial had different results^9^. Presently, there is a paucity of evidence directly comparing neoadjuvant or adjuvant therapies, and the optimal choice among them remains unclear.

When there is a lack of randomized controlled trials (RCTs) directly comparing different treatments, network meta-analysis (NMA) can be used to combine the available evidence and compare the treatments indirectly, under the conditions that heterogeneity and inconsistency are fulfilled^10^. Although NMAs on multimodality treatments of ESCC have been conducted^11,12^, the evidence is still not enough, and new evidence has been presented in the last 2–3 years.

Our study aimed to perform a NMA comparing OS for neoadjuvant and adjuvant CT, CRT, and RT in patients with resectable ESCC.

## Materials and methods

### Literature search

The protocol of this NMA was registered in the PROSPERO database (identification code: 212733).

A systematic search was carried out for available literature published up to July 2018. The searches were limited to articles describing RCTs published in English. RCTs were searched in the EMBASE, MEDLINE, and Cochrane Library databases from 1990. A systematic review of RCTs was conducted following the PRISMA for Network Meta-Analyses (PRISMA-NMA)^13^ and Cochrane guidelines^14^. A combination of “[o]esophageal cancer (or neoplasms)” and the following different terms were used: “squamous cell”, “chemotherapy”, “chemoradiotherapy”, “radiotherapy”, “chemoradiation”, “neoadjuvant”, “adjuvant”, “preoperative”, “postoperative”, and “surgery (or esophagectomy)”. We also used the terms searching for systematic reviews and meta-analyses. References of the articles were also searched for potentially available studies. Abstracts or posters of international meetings such as American Society of Clinical Oncology (ASCO) meetings, European Society for Medical Oncology (ESMO) congresses or Chinese Society of Clinical Oncology (CSCO) meetings were also screened using the abovementioned keywords.

### Study selection

Only full-text articles were included. We focused on treatments for ESCC, and RCTs published in English were included regardless of histology if the following criteria were met: sufficient data of the ESCC subgroup could be obtained or the majority of the patients had ESCC. Patients were confirmed to undergo radical/curative intent resection without distal metastasis. The latest published studies were chosen if there were data published repeatedly.

The studies were excluded in the following situations: non-RCT design, including patients never undergoing surgery, insufficient data for the ESCC subgroup or less than 80% of patients had ESCC.

### Date extraction

Three authors independently reviewed the full text of the enrolled studies. The study endpoint was OS, and the outcome measure was the hazard ratio (HR) with 95% confidence interval (CI). However, HRs and CIs were not available at most times, and they could be estimated using a previously introduced method^15–17^. The HR, 95% CI and standard error of lnHR were carefully extracted from the survival curve.

### Quality of evidence assessment

All enrolled articles underwent quality assessment, and the quality of evidence can be graded to 4 levels: high, moderate, low, and very low^18^. The quality can be downgraded due to publication bias, indirectness, inconsistency or imprecision.

### Risk of bias assessment

All articles were assessed for risk of bias using the Cochrane risk of bias tool for RCTs^19^. Enrolled RCTs were classified into 3 categories: high risk, low risk, or unclear risk. Yongqiang Wang and Lei Shan extracted the data, and Wenhao Zhang verified the data independently.

### Statistical analysis

OS was used as the study endpoint. Standard pairwise meta-analysis was performed for direct comparisons using the inverse variance DerSimonian-Laird random effects model^20^. If a direct comparison was based on 2 or more studies, the study heterogeneity was quantified using the I-squared statistic, and it was considered low, moderate, or high for I-squared values of < 25%, 25% to 50%, and > 50%, respectively^21^. A network of evidence can be constructed in the absence of direct comparisons between treatments^22^. The methods for indirect treatment comparisons are categorized as Bayesian or frequentist^23^. The frequentist method was used in our study, and it was also known as the adjusted indirect treatment comparison (AITC) method, which has been described previously^22,24^. A random effects NMA was carried out with a frequentist setting^23–25^. More information about the research synthesis methods could be obtained from the article^26–28^. The use of Stata in dealing with more complicated networks is introduced in the article^29^, including the evidence for publication bias (as assessed by means of a funnel plot dedicated to network meta-analysis). The adjusted indirect method we also used was introduced by Branko Miladinovic et al.^30^. A heterogeneity parameter (tausquared) was assumed across all comparisons. Each summary effect is presented along with 95% CI and predictive interval. The predictive interval was calculated using the between-study variance tau-squared, providing information on the magnitude of heterogeneity. Contribution matrix is generated during the direct and indirect analysis using Stata 12.0^29^.

Transitivity is one of the key assumptions of NMA^31^; for example, if the information of surgery versus neoadjuvant chemoradiation and surgery versus neoadjuvant chemotherapy is available, then the information of the neoadjuvant chemoradiation versus neoadjuvant chemotherapy comparison can be obtained for the NMA. Usually, the treatment of surgery alone is assumed to be the common treatment as the transitivity assumption and is regarded reasonably consistent among all studies. Participants could be principlely randomized to any of the treatments in the network. Lack of transitivity can present as inconsistency between direct and indirect estimates (loop inconsistency) or between estimates deriving from different study designs (design inconsistency). The inconsistency between direct and indirect estimates or between estimates from different study designs can be studied using the design-by-treatment interaction model^26,32^.

Statistical tests were 2-sided. The statistical analysis and graph drawing were performed with Stata 12.0 (StataCorp LP, College Station, TX, USA).

## Results

### Search results

**Figure 1:**
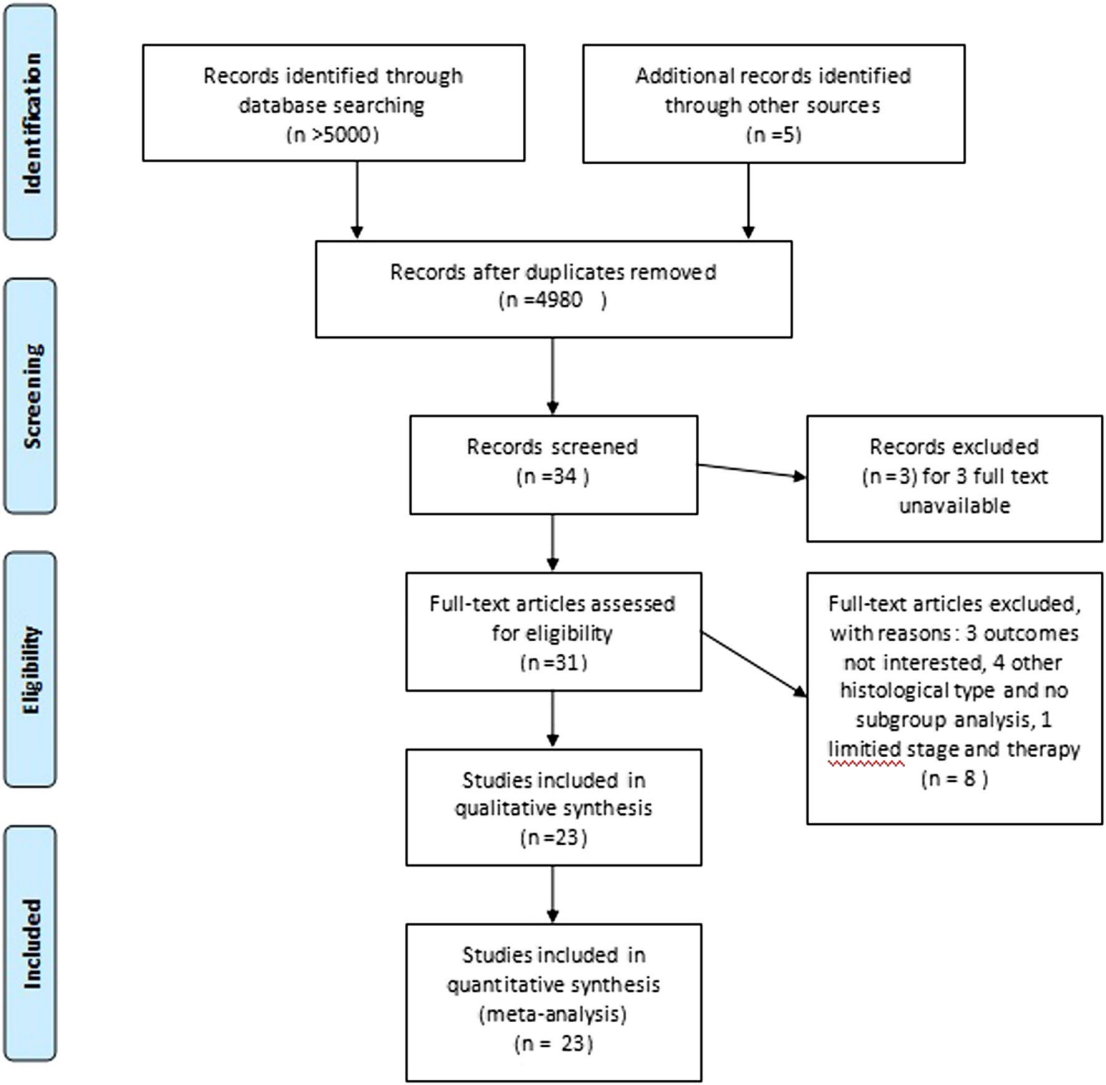
Literature search flow chart.

**Figure 2:**
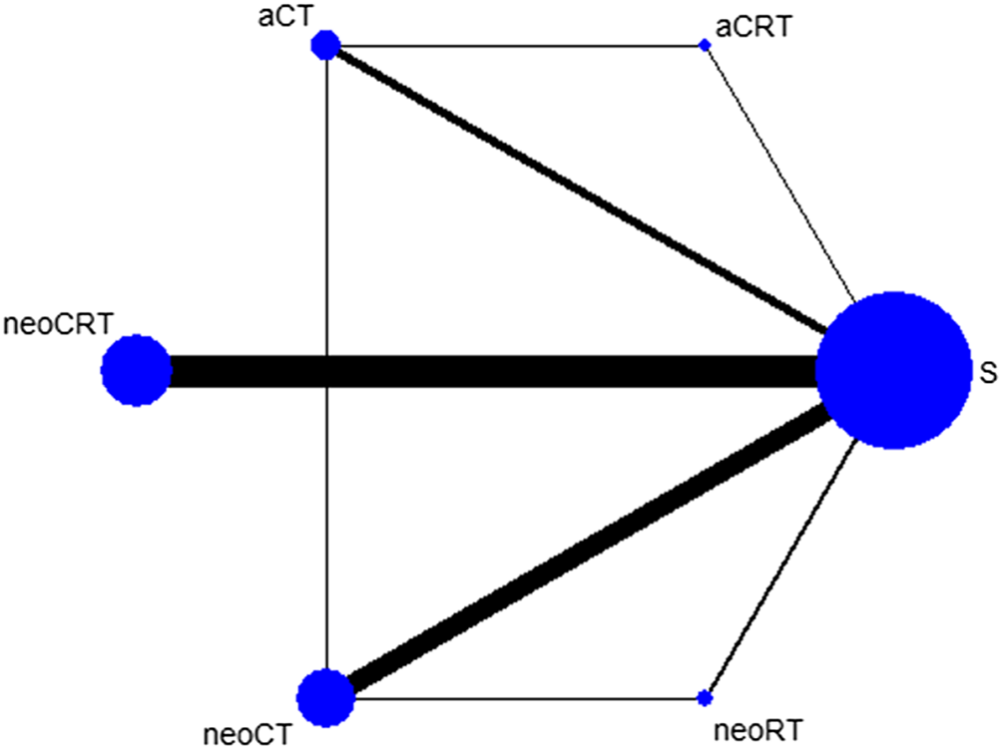
Network plot showing the different treatment modalities. *neoCRT* neoajuvant chemoradiotherapy, *neoCT* neoajuvant chemotherapy, *neoRT* neoajuvant radiotherapy, *aCRT* adjuvant chemoradiotherapy (CRT), *aCT* ajuvant chemotherapy, *S* surgery alone.

More than 5000 articles were identified from the literature search, and 42 potentially eligible articles were retrieved for detailed analysis. In the next step, 14 articles were excluded because of duplicates, the unavailability of the full text, the lack of the outcome of interest, or the lack of subgroup analysis. Twenty-four reports of RCTs^8,33–55^ (published from 1990 to 2018) were intended to be included in the NMA, and 23 RCTs were enrolled after an article from the 2018 CSCO Meeting^55^ was excluded because of limited stage and therapy (only N1 patients were enrolled and there was only one article for adjuvant radiotherapy) (Fig. 1, Supplementary Table 1). Most trials were two-arm trials, and the rest were three-arm trials. The following treatments were compared in the trials: surgery with neoadjuvant CT, surgery with neoadjuvant CRT, surgery with neoadjuvant RT, surgery with adjuvant CT, surgery with adjuvant CRT, neoadjuvant CT with neoadjuvant RT, adjuvant CT with adjuvant CRT, and neoadjuvant CT with adjuvant CT. These are presented in the network plot (Fig. 2).

### NMA results

**Figure 3:**
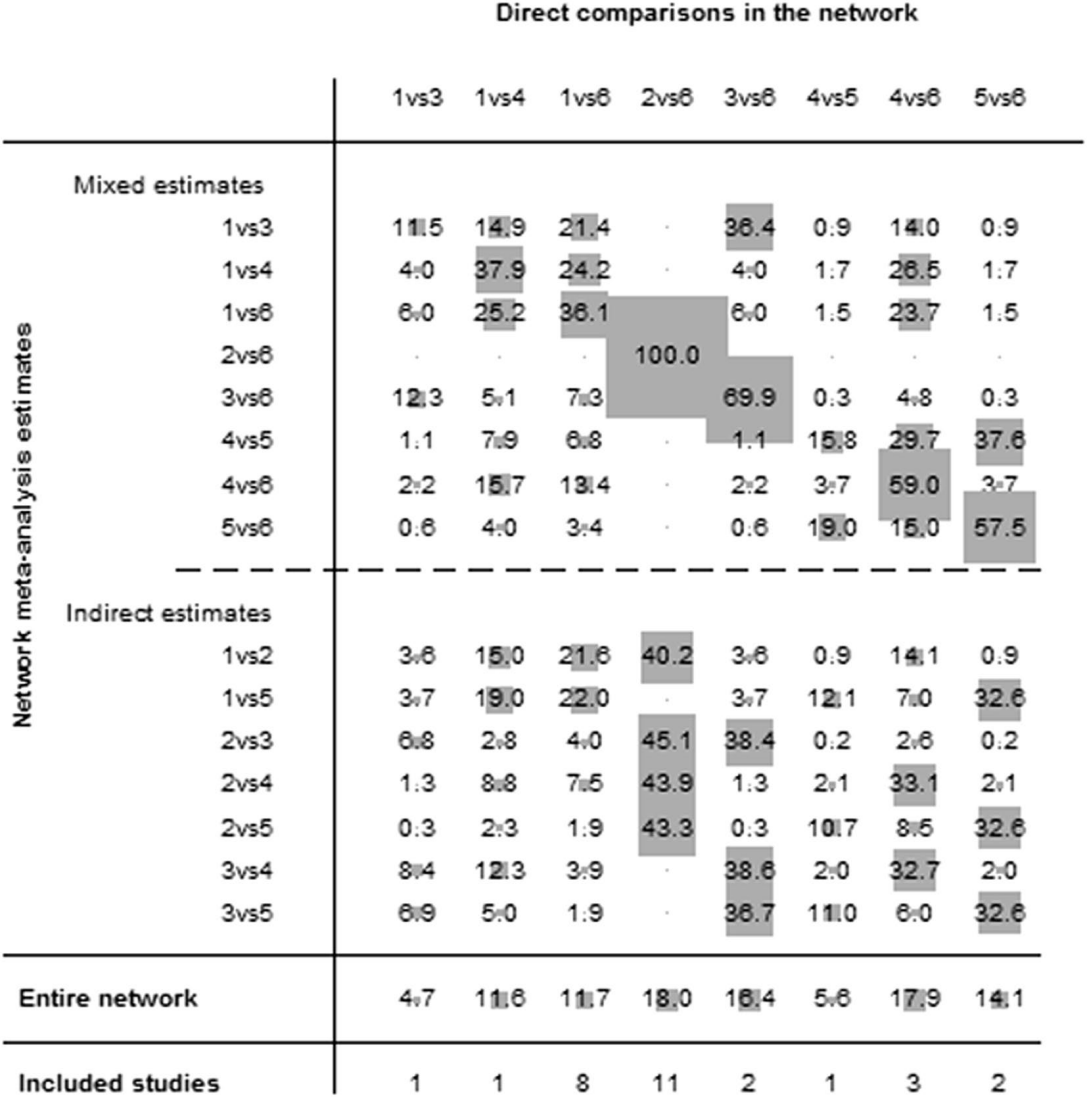
Contribution plot of the direct comparisons and the indirect comparisons. (1: neoadjuvant CT, 2: neoadjuvant CRT, 3: neoadjuvant RT, 4: adjuvant CT, 5: adjuvant CRT, 6:S).

**Figure 4:**
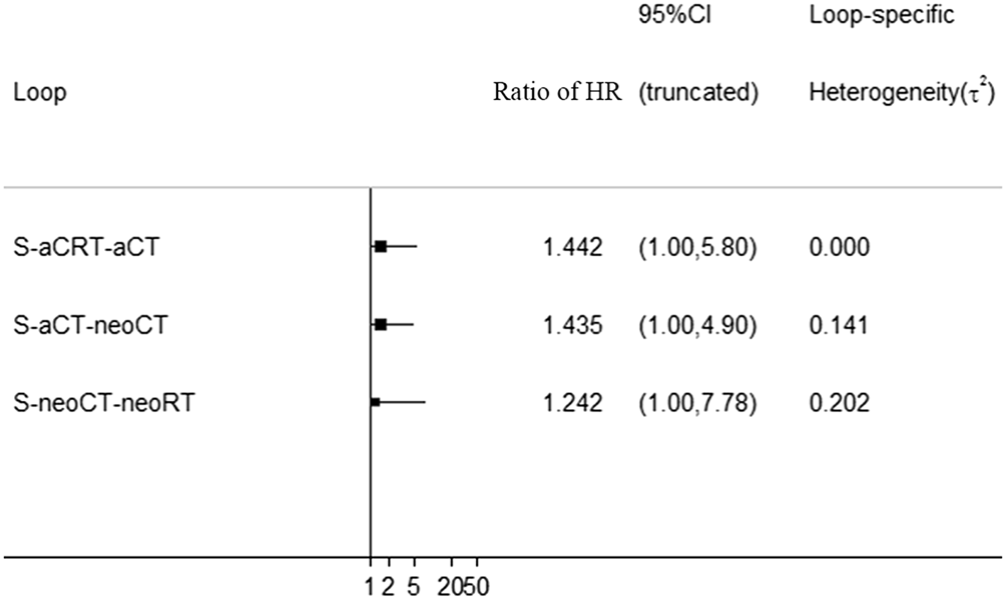
Inconsistency test chart.

**Figure 5:**
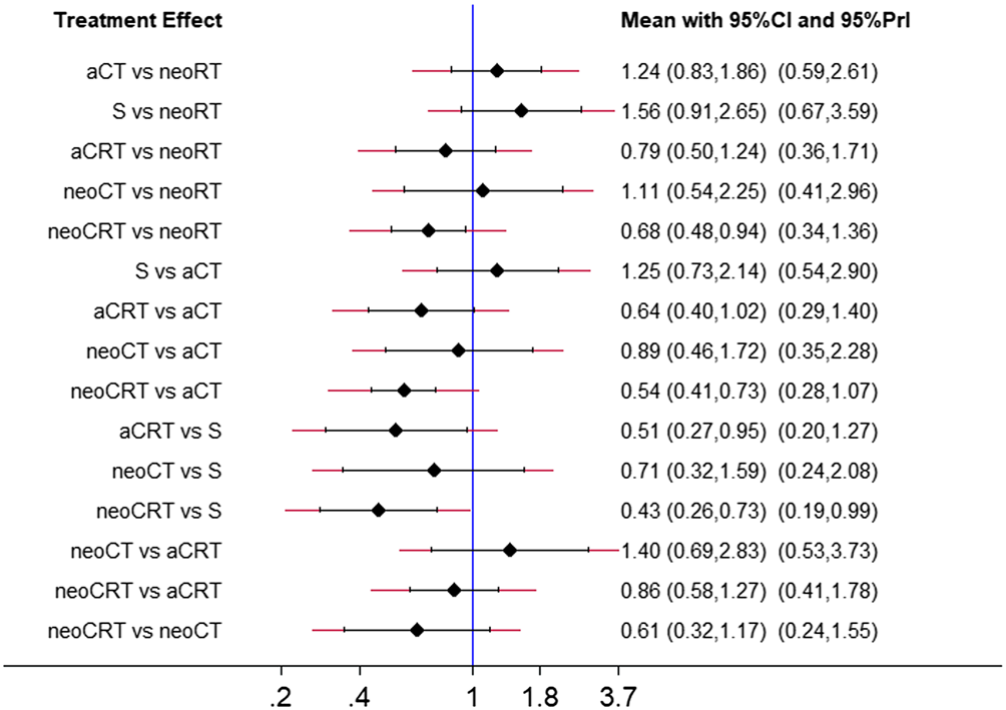
Network meta-analysis results, the treatment effect measure is expressed as hazard ratio.

The network of eligible comparisons for NMA is shown in Fig. 2, and the contribution matrix of the NMA is shown in Fig. 3. In Fig. 3, direct comparisons are represented in the columns of the matrix, and network estimates are represented in the rows of the matrix. Each direct comparison contributes differently to the network summary effects. The matrix is useful to identify the most influential comparisons of the entire network. The inconsistency test chart is shown in Fig. 4. The values of the ratio of two HRs were all close to 1, implying that the direct evidence and the indirect evidence were generally consistent. The network comparisons for OS are shown in Table 1 (Supplementary Table 2). In our model, neoadjuvant CRT, neoadjuvant CT, neoadjuvant RT, and adjuvant CRT offered an OS advantage over surgery alone with HR and CIs showed in Table 1 and Fig. 5. There was also a similar trend for adjuvant CT; however, distinct significance could not be demonstrated (HR 0.80; 0.47–1.36; *P* = 0.401), which indicated that adjuvant CT did not provide a further benefit compared with surgery. There were no significant differences between neoadjuvant CRT and neoadjuvant CT, neoadjuvant CRT and neoadjuvant RT, neoadjuvant CRT and adjuvant CRT, neoadjuvant CRT and adjuvant CT, neoadjuvant CT and neoadjuvant RT, neoadjuvant CT and adjuvant CRT, neoadjuvant CT and adjuvant CT, neoadjuvant RT and adjuvant CRT, neoadjuvant RT and adjuvant CT, and adjuvant CRT and adjuvant CRT.

**Table 1:** Efficacy of all the treatments regimens in network meta-analysis (pooled HR with 95%CI).

neoCRT	1.48 (1.06,2.06)	1.84 (1.38,2.44)	2.30 (1.37,3.85)	1.17 (0.79,1.73)	1.63 (0.86,3.12)
0.68 (0.48,0.94)	neoRT	1.24 (0.83,1.86)	1.56 (0.91,2.65)	0.79 (0.50,1.24)	1.11 (0.54,2.25)
0.54 (0.41,0.73)	0.81 (0.54,1.20)	aCT	1.25 (0.73,2.14)	0.64 (0.40,1.02)	0.89 (0.46,1.72)
0.43 (0.26,0.73)	0.64 (0.38,1.09)	0.80 (0.47,1.36)	S	0.51 (0.27,0.95)	0.71 (0.32,1.59)
0.86 (0.58,1.27)	1.27 (0.81,1.98)	1.57 (0.98,2.52)	1.97 (1.05,3.68)	aCRT	1.40 (0.69,2.83)
0.61 (0.32,1.17)	0.90 (0.45,1.84)	1.12 (0.58,2.17)	1.41 (0.63,3.15)	0.71 (0.35,1.44)	neoCT

The common treatment (surgery) was reasonably consistent across trials, and no evidence of violation of the transitivity assumption was observed. The participants could be randomized to any treatments being compared in the network due to the equal distribution of the modifiers across studies.

### Quality assessment of trials, publication bias and evidence grading

**Figure 6:**
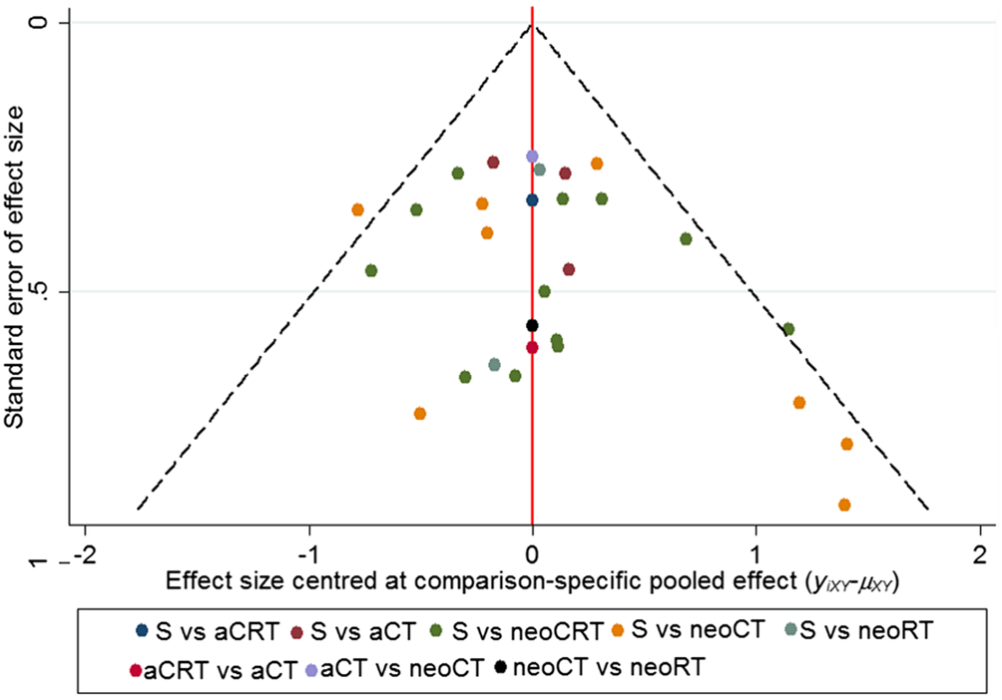
Funnel plot for risk of publication bias.

There was no severe risk of bias in the eligible RCTs (Supplementary Table 3). Funnel plot analysis also did not indicate any prominent risk of publication bias (Fig. 6). These findings, coupled with the absence of inconsistency and the lack of violation of the transitivity assumption, allowed us to grade the RCTs as high or moderate.

## Discussion

ESCC still has a poor 5-year survival, and individualized clinical decisions are difficult to make due to inadequate evidence. High-quality adequately sized trials are few because of the complexity of the surgery and the inconsistency of the clinical decisions made in different medical centers. The potential aggressiveness of this tumor also increases the risk of R1 resection. More reliable evidence is still needed.

Our study analyzed 23 RCTs, and neoadjuvant treatments were demonstrated to be more effective for ESCC. The efficacy in 3636 patients was assessed using OS. Neoadjuvant CRT, neoadjuvant RT, adjuvant CRT and neoadjuvant CT conferred better OS. Adjuvant CT failed to show an OS benefit for ESCC.

As poor outcomes are presented with surgery alone, combined therapies are required. Neoadjuvant therapy has been compared with postoperative therapy or surgery alone for resectable ESCC for curative intent. Stage I patients have not yet been evaluated. The expected advantages of neoadjuvant therapy are as follows. 1. The patients’ response to chemotherapy or radiotherapy can be evaluated through neoadjuvant therapy, and downstaging is expected to make the surgical procedure easier and improve the radical resection rate. 2. Neoadjuvant therapy is expected to kill the micrometastasis, which may be potentially useful to prolong the long-term survival of patients. 3. Patients more easily complete the treatment plan before surgery, and chemotherapeutic drugs can reach the target before the local blood supply is destroyed. Most studies have focused on neoadjuvant CRT and neoadjuvant CT. The recent National Comprehensive Cancer Network (NCCN) guidelines suggest preoperative chemoradiation for patients with stage cT1b-T4a and N0 or N + (RT, 41.4–50.4 Gy + concurrent chemotherapy), and its multidisciplinary recommendation is that combined modality therapy is effective for patients with localized esophageal cancer. Three articles are cited^56–58^; however, the majority of the patients enrolled in the research had adenocarcinoma. A few RCTs that verified the effectiveness of neoadjuvant CRT have been reported since the 1990s; though few studies contributed to OS, the pathologic complete response (pCR) rate was higher in these patients. A high-quality study by Bosset et al.^40^ reported that recurrence-free survival was significantly improved in patients with squamous cell carcinoma with no benefit of OS. The patient characteristics, including histological type and stage, and CRT protocols varied greatly in the meta-analysis performed in North America and Europe, and radical surgery was regarded as a vital factor for long-term survival. Guidelines for the diagnosis and treatment of carcinoma of the esophagus in Japan^59^ recommended neoadjuvant chemotherapy with cisplatin + 5-fluorouracil (5-FU) in patients with resectable stage II or III thoracic esophageal carcinoma according to JCOG9204^43^ and JCOG9907^8^. Postoperative irradiation was only suggested for use when there was local residual tumor or local recurrence. However, few cases presented pCR^60^, and few meta-analyses demonstrated its survival benefit over surgery alone^61^. The role of neoadjuvant RT remains unsatisfactory, and its impact can be affected by several elements, such as the volume of irradiation, daily fractions, total dose, and interaction with other treatments. It has been reported that daily fractionation > 40 Gy with radiation could increase the late toxicity risk^62^, and as a result, it may be harmful to long-term survival. The value of neoadjuvant RT remains controversial, and no recommendation has been made in the clinical guidelines until now.

The value of postoperative adjuvant therapy for stage I patients has not yet been studied. The expected advantages of postoperative adjuvant therapy are to address the residual tumor, lymph node metastasis, and possible micrometastasis. However, disadvantages also existed, as no visible target could be used to evaluate the effect of postoperative adjuvant therapy and lower compliance compared with that of neoadjuvant therapy. More evidence suggests better survival benefits for neoadjuvant therapy than for adjuvant therapy^63,64^.

Our analysis showed that neoadjuvant CRT may have the best OS benefit and seems to significantly improve OS compared with surgery alone (HR = 0.43,95% CrI 0.26–0.73, *P* = 0.008). This may be due to four mechanisms: 1. radiation damage can be enhanced by cell repair inhibition; 2. radioresistant-phase tumor cells can be cleared and radiosensitive-phase cells can be accumulated; 3. hypoxic cells can be cleared; and 4. the repopulation of tumor cells can be inhibited^65^. Theoretically, chemotherapy can control micrometastatic tumor cells, and radiotherapy can control regional tumors; therefore, the effect of spatial cooperation was presented. Sometimes it can lead to downstaging with a higher radical resection rate, which may improve OS^66^. Nonetheless, it could not be denied that neoadjuvant CRT was also associated with a higher risk of postoperative mortality than neoadjuvant CT or surgery alone^67^. As mentioned before, treatment outcomes differ according to the individual cases, the administration of the drug, the dosage of the radiation and so on. Additional high-quality randomized trials with larger sample sizes are needed.

In our study, we also suggest that neoadjuvant CT had a survival benefit over surgery alone (HR = 0.710, 95% CI 0.32–1.59, *P* = 0.009); however, a statistically significant survival benefit of neoadjuvant CRT compared with neoadjuvant CT could not be demonstrated in our study (HR = 0.61, 95% CI 0.32–1.17, *P* = 0.08). The result was similar to that of Sjoquist et al.’s study^63^. Neoadjuvant CT was reported to have a survival benefit over surgery, but they were not able to demonstrate a statistically significant survival benefit for neoadjuvant CRT against neoadjuvant CT after enrolling patients with both squamous cell carcinoma and adenocarcinoma (2049 patients and 1291 patients, respectively). It is always difficult to conduct a direct comparison of neoadjuvant CRT against neoadjuvant CT because of the limited sample size, and our NMA seemed to provide some of the latest evidence for the multiple-disciplinary treatment for patients with ESCC.

Several considerations should be kept in mind about our study. Single-patient data could not be obtained, and the survival data were extracted from the survival curve. Some errors may exist compared with the original data. Patients with adenocarcinoma were included in one of the studies enrolled (with 137 ESCC patients and 57 adenocarcinoma patients), and there was no subgroup analysis^53^. Some of the trials were found to have a high or unclear risk of bias, though the test of heterogeneity and inconsistency was negative. The studies included crossed nearly three decades, over which the CT drugs, implementation of radiotherapy, or even the surgery procedure changed considerably, and OS might be affected in different studies. The staging system and technology have advanced, and more sensitive disease staging is provided; inevitably, the distribution of stage varied greatly among the studies in different periods. This may affect the determination of the evaluation of the survival benefit. Although the AITC method we used has been found to be more favorable to the Bayesian method because of its simplicity considering that direct treatment comparisons are absent and networks are less complex^68^, the AITC method cannot handle the correlations well in multiarm trials. Therefore, our study cannot totally substitute high-quality multicenter, prospective, randomized controlled clinical trials directly comparing the different treatments, and readers should interpret the results with caution.

In summary, regional variability exists in the use of multimodality therapy in patients with ESCC, neoadjuvant CRT is preferred in Europe and the United States, neoadjuvant CT is recommended in Asian countries, and there is no clear recommendation in China due to the controversy. Our NMA suggests that neoadjuvant CRT or neoadjuvant CT may prolong survival in patients with ESCC. Individualized molecular markers may provide some information for clinical decisions in the future.

## Supplementary Information

Supplementary Legends.

Supplementary Table 1.

Supplementary Table 2.

Supplementary Table 3.
