# Supplementary material for: A network meta-analysis for neoadjuvant and adjuvant treatments for resectable squamous cell carcinoma of esophagus
Source: Sci Rep. 2021 Mar 24;11:6800. doi: 10.1038/s41598-021-86102-8 (PMC7990939; doi:10.1038/s41598-021-86102-8)
Supplement: Supplementary file 1 — Supplementary Legends. [file 41598_2021_86102_MOESM1_ESM.doc]

**Supporting Table Legends**

Supplementary Table 1 The articles enrolled

Supplementary Table 2 HR ln_HR se_ln_HR retrieved and Heterogeneity

Supplementary Table 3 Risk of bias
