# Supplementary material for: A network meta-analysis for neoadjuvant and adjuvant treatments for resectable squamous cell carcinoma of esophagus
Source: Sci Rep. 2021 Mar 24;11:6800. doi: 10.1038/s41598-021-86102-8 (PMC7990939; doi:10.1038/s41598-021-86102-8)
Supplement: Supplementary file 4 — Supplementary Table 3. [file 41598_2021_86102_MOESM4_ESM.doc]

**Supplementary table 3.** Risk of bias of included studies calculated according to the Cochrane Risk of Bias tool.

| **Study** | **Adequate Sequence Generation (Selection Bias)** | **Allocation Concealment (Selection Bias)** | **Blinding of Participants and Researchers (Performance Bias)** | **Blinding of Outcome Assessment (Detection Bias)** | **Incomplete Outcome Data (Attrition Bias)** | **Selective Reporting (Reporting Bias)** | **Other Bias** |
| --- | --- | --- | --- | --- | --- | --- | --- |
| **Kelsen 1990** | + | ? | ? | ? | + | + | + |
| **Schlag 1992** | + | + | ? | ? | + | ? | + |
| **Nygaard 1992** | + | + | ? | ? | + | ? | + |
| **Maipang 1994** | + | ? | ? | ? | + | ? | + |
| **Le Prise 1994** | + | + | ? | ? | + | ? | + |
| **Pouliquen 1996** | ? | ? | ? | ? | + | ? | + |
| **Ando 1997** | + | + | ? | ? | + | ? | + |
| **Bosset 1997** | ? | + | ? | ? | + | ? | + |
| **Law 1997** | ? | + | ? | ? | + | ? | + |
| **Ancona 2001** | + | + | ? | ? | + | + | + |
| **Ando 2003** | + | ? | ? | ? | + | ? | + |
| **Tachibana 2003** | ? | ? | ? | ? | + | ? | + |
| **Lee 2004** | ? | ? | ? | ? | + | ? | + |
| **Burmeister 2005** | + | + | ? | ? | + | ? | + |
| **Natsugoe 2006** | + | + | + | ? | + | ? | + |
| **Allum 2009** | ? | ? | ? | ? | + | ? | + |
| **Lv 2010** | + | ? | ? | ? | ? | ? | + |
| **Cao 2009** | + | ? | ? | ? | ? | ? | + |
| **Boonstra 2011** | + | + | + | ? | + | ? | + |
| **Van Hagen 2012** | + | + | ? | ? | + | ? | + |
| **Ando 2012** | + | ? | ? | ? | + | ? | + |
| **Mariette 2014** | + | + | ? | ? | + | ? | + |
| **Shapiro 2015** | + | + | + | ? | + | ? | + |

Heterogeneity parameter tau using stata

Side Direct Indirect Difference tau

Coef. Std. Err. Coef. Std. Err. Coef. Std. Err. P>|z|

A B .7816152 .2690449 -.1516025 .215446 .9332177 .3392657 0.006 0.0669255

A C .6776303 .2577017 -.7695662 .5349934 1.44719 .5945693 0.015 0.1344288

A D -.4752058 .3789353 -.0834147 .2980517 -.391791 .4821066 0.416 0.2856339

A F -.413318 .1985319 -.2815883 .3658055 -.131729 .4106905 0.748 0.2969435

B C -.0736185 .3034198 .8764712 .4620639 -.9500896 .5580141 0.089 0.2094392

B E-.0581382 .4331016 -.2100937 .5650045 .1519556 .7108821 0.831 0.290277

B F -.5546491 .1300644 -1.891362 .5504324 1.336713 .5627329 0.018 0.134428

C F-.7636633 .3440036 -.9511825 .4470969 .1875191 .5704556 0.742 0.2901195

D E .4418328 .6681143 .2922532 .4369586 .1495795 .7983167 0.851 0.2863893

D F -.2719341 .2451523.1087268 .3678278 -.3806609 .4416105 0.389 0.2806912

E F -.3647901 .4357639 -.6786626 .5319088 .3138724 .6897076 0.649 0.2854294

A：neoRT B: aCT C: S D: aCRT E: neoCT F: neoCRT
